# Misoprostol as an adjunct to oxytocin can reduce postpartum-haemorrhage: a propensity score–matched retrospective chart review in Bamenda-Cameroon, 2015–2016

**DOI:** 10.1186/s12884-019-2407-3

**Published:** 2019-07-22

**Authors:** Frederick Morfaw, Mercy Fundoh, Christopher Pisoh, Bi Ayaba, Lawrence Mbuagbaw, Laura N. Anderson, Lehana Thabane

**Affiliations:** 1 Department of Health Research Methods, Evidence and Impact, McMaster University, Hamilton, ON Canada; 2 Department of Obstetrics and Gynaecology, Faculty of Medicines and Biomedical Sciences University of Yaounde 1, Yaoundé, Cameroon; 3 Department of Clinical Sciences, Faculty of Health Sciences, University of Bamenda, Bamenda, Cameroon; 4 Regional Hospital Bamenda, Bamenda, Cameroon; 5 Biostatistics Unit, St Joseph Healthcare—Hamilton, Hamilton, ON Canada; 6 Centre for Development of Best Practices in Health, Yaoundé Central Hospital, Yaoundé, Cameroon

**Keywords:** Post-partum haemorrhage, Misoprostol, Oxytocin, Maternal mortality

## Abstract

**Background:**

There is some evidence that suggests misoprostol may supplement the action of oxytocin in preventing post-partum haemorrhage (PPH). The primary objective of this study was to determine the effect of the administration of 600 μg misoprostol in addition to oxytocin versus oxytocin alone, on the risk of PPH among pregnant women after delivery. The secondary objectives were to determine the effects of the above combination on maternal death and blood transfusion among pregnant women after delivery; and to determine the incidence of PPH, its case fatality, and the maternal mortality ratio in our hospital.

**Methods:**

*Design and setting*: Retrospective chart review of 1736 women delivering at the Regional Hospital Bamenda Cameroon, between 2015 and 2016. This was a pre versus post study following a policy change in the prevention of PPH.

*Exposure groups*: One group received oxytocin-misoprostol (January–April 2016: period after policy change), and the second group received oxytocin-only (January–April 2015: period before policy change) after delivery.

*Outcomes*: The primary outcome was PPH, and the secondary outcomes were maternal death and blood transfusion.

*Statistical analysis*: A 1:1 matching with replacement was done with the propensity score (PS). The groups were compared using PS matching with conditional logistic regression on the matched pairs as the main analysis. A sensitivity analysis was done using other PS adjustment methods and multiple regression.

**Results:**

Of the 1736 women included in this study, 1238 were matched and compared. Women who received oxytocin-misoprostol were less likely to have PPH as compared to those receiving oxytocin-only (odds ratio [OR] 0.22, 95% confidence interval [CI] 0.08, 0.59, *p* = 0.003). This reduced odds of PPH was upheld in the different sensitivity analyses. There were no significant differences in the odds of maternal death and the use of blood transfusions between the two groups: OR 3.91, 95% CI [0.44, 35.08], *p* = 0.22, and OR 0.89, 95% CI [0.14–5.63], *p* = 0.91, respectively. Sensitivity analyses showed similar results. The incidence of PPH was 2.9% (before adding misoprostol the incidence was 4.4% and after adding misoprostol it was 1.5%), the case fatality rate of PPH was 1.96%, and the overall maternal mortality ratio in the hospital was 293 maternal deaths/100000 life births.

**Conclusion:**

Our evidence suggests that using 600 μg misoprostol as an add-on to oxytocin in the prevention of post-partum haemorrhage significantly reduces the odds of PPH without affecting other maternal outcomes.

## Background

Post-partum haemorrhage (PPH) refers to bleeding from the genital tract greater than or equal to 500 cc following vaginal delivery, or greater than or equal to 1000 cc following a caesarean section [1]. PPH is responsible for one maternal death every 4 min in low-income countries [2]. Most of these maternal deaths occur within the first 24 h following delivery, but can largely be prevented by the use of prophylactic uterotonics after delivery [3]. This is because PPH is primarily due to uterine atony [4].

Active management of the third stage of labour (AMTSL) is an evidence-based intervention which is recommended for all deliveries to prevent PPH [3]. The administration of a prophylactic uterotonic agent is a key component of AMTSL, and the World Health Organisation (WHO) recommends the use of oxytocin (10 IU, intra venous/intra-muscular) as the uterotonic drug of choice [3]. Despite the widespread use of oxytocin in AMTSL, the high rates of PPH observed in low-income countries is indicative of the fact that this strategy may be lacking in certain aspects, thus justifying the need for add-ons to supplement the uterotonic properties of oxytocin.

One of such potential add-ons is misoprostol which is a prostaglandin E1 analogue with strong uterotonic properties [5]. Given its established safety profile in obstetrics, misoprostol is recommended by WHO as an alternative to oxytocin for the prevention of PPH in settings where the latter is unavailable [3]. Given their independent pathways of action, one could expect a synergistic effect of both drugs when used in combination to prevent PPH [6]. There is strong biological evidence suggesting that misoprostol can augment the effectiveness of injectable uterotonic agents such as oxytocin used in AMTSL [7].

Since October 2015, following a drug donation of misoprostol from the Life for African Mothers Non-Governmental Organisation, there was a policy change in the preventive management of PPH in the Regional Hospital Bamenda (RHB) Cameroon, from the use of oxytocin-only, to the use of an oxytocin-misoprostol combination. Consequently 600 μg misoprostol was routinely given in the immediate post-partum period, either orally or rectally, as an add-on to oxytocin in AMTSL, to all women who delivered in the maternity of the Regional Hospital Bamenda, to help in the prevention of PPH. This policy change was based more on biological plausibility than evidence. No study has been done to evaluate the effect of this combination on the prevention of PPH, and consequently the reduction of maternal mortality. It is important to note that the drug donation continues till date, and there are plans for the hospital administration to ensure the continuity of the supply once the donation comes to an end.

The primary objective of this study was therefore to determine whether the routine administering of 600-μg misoprostol to pregnant women after placental delivery in addition to routine AMTSL using oxytocin, was associated with reduced risk of PPH after adjusting for potential confounders.

Our secondary objectives were to determine whether this routine administration of 600-μg misoprostol was associated with reduced risk of maternal death and blood transfusion after adjusting for potential confounders. In addition, we sought to determine the incidence of PPH at the Regional Hospital Bamenda as well as its case fatality rate; and to determine the causes of PPH at the Regional Hospital Bamenda. We equally sought to estimate the maternal mortality ratio at the Regional Hospital Bamenda, and to describe the associated causes of maternal death.

Our research hypothesis was that the use of 600 μg misoprostol as an add-on to standard care with oxytocin for AMTSL is protective against PPH, maternal mortality and use of blood transfusions among women after delivery.

## Methods

### Study type

This was a retrospective chart review study.

### Setting

This was a hospital-based study conducted at the maternity of the Regional Hospital Bamenda (RHB) in the North West Region of Cameroon. Cameroon is a sub-Saharan West African country. It has an estimated population of about 24 million inhabitants [8]. The Regional Hospital Bamenda is the lone referral hospital in Bamenda the capital of the North West Region of Cameroon, and serves a population of about 337,036 inhabitants [9]. In this hospital, about 3,360 women give birth annually [9].

### Inclusion and exclusion criteria

We included in our study pregnant women who delivered at the maternity of the Regional Hospital Bamenda at a gestational age of 20 weeks or more, and who had complete case records on the evolution of labour from the moment of admission until discharge from the hospital.

We excluded women with incomplete case records during the study period, those with medical records which had been physically damaged, women who delivered before arrival to the hospital. We equally excluded women who had delivered elsewhere and developed PPH, and were then referred for the management of PPH to the Regional Hospital Bamenda.

### Study period

**Fig. 1:**
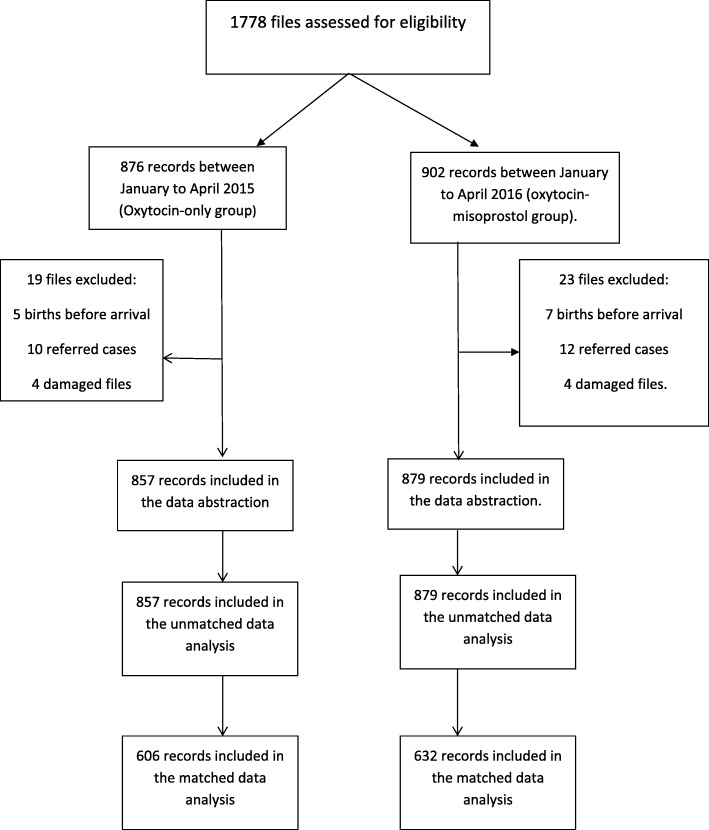
Flow chart of study participants. Summarises the flow of study participants within the study

Data for this study was collected between November 2017 to March 2018. Given that the gift of misoprostol was received in October 2015, we could not have a concurrent comparison group, as the drug was routinely administered to all women thereafter. Our comparison group was therefore drawn from a historical cohort of women delivering in the same hospital within a similar time frame (see Fig. 1). We used a 4-month period within each time frame, that is, from January to April 2015 (period of no misoprostol use), and from January to April 2016 (period of routine misoprostol use). These time frames were chosen to minimise any biases due to differential staffing because during these periods the maternity staff was the same (same 4 Obstetrician Gynaecologists, same midwives in the labour room and postnatal wards, same staff in the theatre). To the best of our knowledge, the quality of care received by the pregnant women during these periods was about the same, and only differed in terms of the routine administration of misoprostol. There were no other changes to clinical practice for PPH over this period.

### Study variables

The intervention was the use of misoprostol in addition to standard care for AMTSL. During the period when it was available, 600 μg misoprostol was given either orally or rectally to all women who delivered in the maternity of the Regional Hospital Bamenda, irrespective of the mode of delivery. The study group receiving this intervention was referred to as the oxytocin-misoprostol group.

The comparator was standard active management of the third stage of labour using oxytocin-only as the uterotonic drug. The study group receiving this was referred to as the oxytocin-only group.

### Study outcomes

The primary outcome measure was PPH defined as vaginal blood loss ≥500 cc within the first 24 h following vaginal delivery or blood loss ≥1000 cc following a caesarean section [1]. However rigorous measurement of blood loss was not routinely done in our maternity. For the purpose of this study, we identified potential cases of PPH from diagnoses recorded in the hospital charts. These diagnoses of PPH were based solely on the clinical judgment of the team on duty who made the diagnosis. The records of these potential PPH cases were reviewed by an adjudication team of two obstetrician gynaecologists involved in the routine care of women within the facility. This team used information noted in the records such as reports of PPH, estimates of blood loss after delivery, occurrence of hemodynamic shock after delivery, laboratory results of haemoglobin level before and after deliver as well as the use of blood transfusion after deliver, in order to adjudicate whether the primary outcome (PPH) had occurred. The adjudication team was blinded to the treatment option used in each case. PPH was recorded as a dichotomous variable.

Secondary outcome measures included maternal death defined as “the death of a woman whilst pregnant or within 42 days of delivery or termination of pregnancy, from any cause related to, or aggravated by pregnancy or its management, but excluding deaths from incidental or accidental causes” [10]; blood transfusions following delivery; the incidence and causes of PPH; the maternal mortality ratio and causes of maternal death. In addition, we tried to collect information on side effects of misoprostol (such as shivering; fever > 38 °C; diarrhoea, nausea, vomiting), but these were not routinely noted in the files and thus these outcomes could not be evaluated.

### Confounders

Based on a literature review, greater maternal age, higher gravidity, higher parity, a maternal history of postpartum haemorrhage, induced labour, multiple pregnancy, foetal macrosomia, polyhydramnios, increased duration of labour, and delivery by caesarean section are a selection of potential confounders of the relationship between the method used for the prevention of PPH and the occurrence of PPH, and may be associated with increased risk of PPH [11, 12]. Data was collected on these confounders when possible, and adjustments made for them by matching or during analysis.

### Data collection

Baseline demographic data as well as outcome related data was collected on a standardized pretested data abstraction form. The form had no personal identifier other than the numbers assigned during data abstraction. Data was collected from the files of the women, and supplemented where necessary with hospital records from the labour room, wards and theatre registers. The need for individual patient consent for data collection was waived by Bamenda Regional Hospital Institutional Review Board.

The collected data was transferred into a Microsoft Excel version 13 spread sheet on an independent and secure computer where it was checked for accuracy and completeness. These data were eventually transferred to the Statistical Package for Social Sciences (SPSS) software version 20.0 [13] for analysis. Propensity score matching was done using the R software (version 3.51) [14].

### Statistical methods

#### Creating the propensity score model

It has been noted that in observational studies, there is a tendency for the existence of systematic differences in baseline characteristics between treated and untreated subjects, and it is important to account for such differences when estimating treatment effects [15]. In recent times, the propensity score is increasingly being used as a balancing score to minimise such differences [15]. The propensity score represents the estimated conditional probability of being assigned to either of the treatment groups given the patients’ pre-treatment characteristics [16]. We therefore opted to use it in our analysis to balance differences in baseline characteristics between the oxytocin-misoprostol group and the oxytocin-only group.

Our propensity score matching model was created using the Coarsened Exact Matching method with replacement [17]. This method was chosen because amongst the multiple methods of matching which we tried out, it resulted in the lowest standardised mean differences between treatment groups for the different matched variables. We did a one-to-one matching with replacement given that it is appropriate in cases where the treatment group is bigger than the control group [18]. Furthermore, it provides more unbiased treatment effect estimates relative to a matching without replacement [18]. For the matching, we did an automated coarsening, and this provided us with a sufficiently large matched sample.

For the matching, the grouping variable was the use of misoprostol or not, while the covariates matched for were age in years, gravidity, parity, history of delivery of a macrosomic baby in the previous pregnancy, whether or not the woman was referred from a different facility, whether or not labour was induced, the mode of delivery and the birth weight of the child. These variables were selected among available baseline covariates based on known associations between these and PPH [11, 12].

#### Comparison of the baseline characteristics

The characteristics of the study participants in the two study groups, was described using descriptive statistics reported as count (percentage) for categorical variables, and mean (standard deviation [SD]) or median (first quartile, third quartile) for continuous variables depending on the distribution. We directly compared the baseline characteristics between our two treatment groups before and after matching. For each variable compared, we used an absolute standardized mean difference threshold of less than 10% as proof of balance between the groups [15].

#### Analysis of primary outcome

We conducted a propensity score (PS) matched data analysis using conditional logistic regression based on 1:1 matched samples. The use of conditional logistic regression in analysing matched case-control data is increasingly being used as a standard procedure [19], hence the reason we choose it as our primary analysis method.

We conducted sensitivity analyses with the other PS-adjustment methods, multivariable logistic regression analysis and analysis on the unmatched data. Our rationale for using the other PS-methods was that there are several ways of using the PS to adjust for confounding [15], each of them worthy of exploration. These methods are stratification on the propensity score, inverse probability of treatment weighting (IPTW) using the propensity score, and covariate adjustment using the propensity score [15]. The rationale for the multivariable analysis comparison was because of the fundamental differences between PS-methods and multivariable regression approaches, with propensity score analysis modelling the relationship between the covariate and the putative cause, while regression adjustment models the relationship between the covariate and the outcome [20]. However, research evidence suggests that the use of multivariable analysis leads to similar results when compared to propensity-score adjusted approaches [21]. Our hypothesis therefore was that the results would remain robust under all these methods.

#### Analysis of secondary outcomes

We conducted a PS matched data analysis using conditional logistic regression based on 1:1 matched samples as the main analysis. A sensitivity analysis was done using the same approaches as with the primary outcome, and for the same reasons.

We presented the results as odds ratio (OR), corresponding 95% confidence interval (CI) and associated *p*-values for each outcome. All of our statistical analyses were performed using a 2 tailed test, and the level of statistical significance was set at 0.05.

## Results

### Baseline characteristics and assessment of the matching

A total of 1736 women were included in this study. Of these 1736 women, 1238 were matched. Table 1 summarises the characteristics of study participants in the unmatched and matched study populations for the two treatment groups (oxytocin-only vs oxytocin-misoprostol).

**Table 1:** Characteristics of study participants in the unmatched and matched study populations for oxytocin-only vs oxytocin-misoprostol (Coarsened exact method matching)

	Unmatched population (*n* = 1736)			Matched Population (*n* = 1238)		
	Oxytocin-only (*n* = 857)	Oxytocin-misoprostol (*n* = 879)	Standard difference,%	Oxytocin-only (*n* = 606)	Oxytocin-misoprostol (*n* = 632)	Standard difference,%
Age in years, Mean (SD)	26.31 (5.17)	26.25 (5.08)	1.1	25.23 (4.44)	25.19 (4.27)	0.9
Gravidity, Mean (SD)	2.58 (1.51)	2.57 (1.55)	0.9	2.25 (1.19)	2.20 (1.21)	3.6
Parity, Mean (SD)	1.29 (1.32)	1.29 (1.37)	0.3	1.09 (1.09)	1.07 (1.23)	2.1
History of macrosomic baby n (%)	94 (11.0)	62 (7.10)	13.7	47 (7.8)	37 (5.9)	7.6
Patient referred n (%)	27 (3.20)	20 (2.30)	5.4	3 (0.5)	3 (0.5)	0.3
Induction of labour in indexed pregnancy n (%)	30 (3.50)	45 (5.10)	8.0	3 (0.5)	5 (0.8)	3.7
Mode of delivery caesarian section n (%)	125 (14.6)	83 (9.4)	15.9	46 (7.6)	36 (5.7)	7.6
Birth weight of babies in the indexed pregnancy in grams, Mean (SD)	3393.9 (516.7)	3182.6 (481.1)	42.3	3362.42 (419.83)	3230.66 (412.77)	31.7

*SD* Standard Deviation

Comparison of the standardised mean differences between the variables in the unmatched and matched data samples shown in Table 1 indicate an overall lower standardised mean difference in the matched data. The balance was successful for all the covariates except for the birth weight of the babies, for which we did not achieve balance.

### Post-partum haemorrhage

**Fig. 2:**
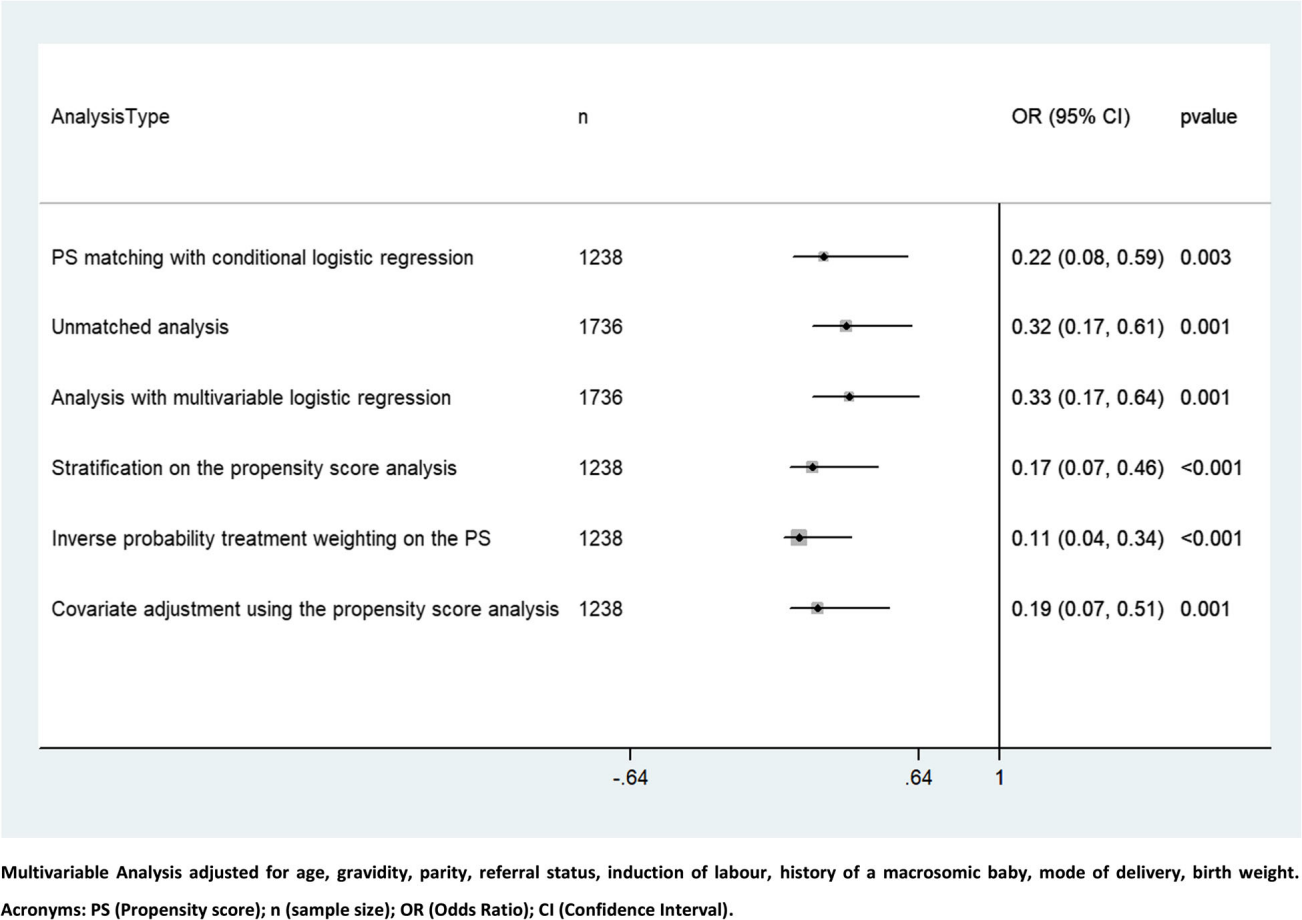
Comparing the odds of post-partum haemorrhage between oxytocin-misoprostol vs oxytocin only using different methods of analysis (Main analysis and sensitivity analysis)

In the unmatched data, PPH was recorded in 1.5% (13/879) of women in the oxytocin-misoprostol group, and in 4.4% (38/857) of women in the oxytocin-only group. In the matched data, PPH was recorded in 0.8% (5/632) of women in the oxytocin-misoprostol group, and in 4.3% (26/606) women in the oxytocin-only group. Figure 2 summarises the odds of PPH between the two treatment groups using different methods of analysis. Women who received oxytocin-misoprostol were less likely to have PPH as compared to those receiving oxytocin-only (main analysis, OR 0.22, 95% CI 0.08, 0.59, *p* = 0.003). This reduced odds of PPH was upheld in the different sensitivity analyses (Fig. 2).

### Maternal deaths

In the unmatched data, there were 4 (0.45%) maternal deaths among the 879 women in the oxytocin-misoprostol group, and 1 (0.12%) among the 857 women in the oxytocin-only group. Table 2 summarises the risk of maternal death between the two treatment groups. There was no significant difference in the odds of maternal death between the two treatment groups (OR 3.91, 95% CI [0.44, 35.08]). This result is presented for the unmatched data only as none of the cases of maternal death were matched, hence a propensity score analysis was not possible with this variable (Table 2).

**Table 2:** Association between maternal death and the type of drug used for PPH prevention

Main Analysis	Odds ratio (95% CI)	*p*-value
Unmatched analysis (*n* = 1238)	3.91 (0.44–35.08)	0.223
Analysis with multivariable logistic regression on the unmatched data (*n* = 1238) ^a^	5.32 (0.095–298.58)	0.416
Analysis with the matched data was not conducted as there were no cases of maternal death in the matched data		

^a^Analysis adjusted for age, gravidity, parity, referral status, induction of labour, history of a macrosomic baby, mode of delivery, birth weight

### Blood transfusion

**Fig. 3:**
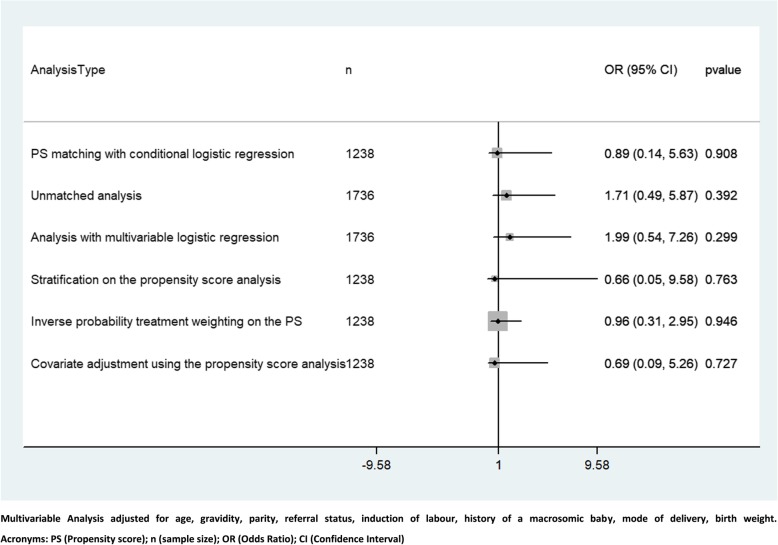
Comparing the odds of blood transfusion between oxytocin-misoprostol vs. oxytocin only using different methods of analysis (Main analysis and sensitivity analysis)

In the unmatched data, 7 of the 879 women (0.79%) in the oxytocin-misoprostol group, and 4 of the 857 women (0.46%) in the oxytocin-only group received a blood transfusion. In the matched data, 2 of the 632 women (0.32%) in the oxytocin-misoprostol group, and 3 of the 606 (0.49%) women in the oxytocin-only group received a blood transfusion. Figure 3 summarises the odds of blood transfusion between the two treatment groups. There was no significant difference in the odds of blood transfusion between the two treatment groups in both the main and sensitivity analysis (main analysis, OR 0.89, 95% CI [0.14–5.63]) (Fig. 3).

### Incidence and causes of PPH

Amongst the 1736 women included in the study, there were 51 cases of PPH, giving an overall incidence of PPH of 2.9%. The incidence before the intervention was 4.4% (38/819) and the incidence after the intervention was 1.5% (13/866). Table 3 summarises the causes of PPH in the two study groups. The most common cause of PPH identified in both groups was uterine atony (50% of identified causes).

**Table 3:** Causes of Postpartum haemorrhage in the two treatment groups

	Oxytocin-only group (*N* = 20): n (%)	Oxytocin-Misoprostol group (*N* = 10): n (%)	Total^a^ (*N* = 30): n (%)
Uterine atony	9 (45.0)	6 (60.0)	15 (50.0)
Retained placental tissue	6 (30.0)	1 (10.0)	7 (23.3)
Genital laceration	5 (25.0)	2 (20.0)	7 (23.3)
Coagulopathy	0 (0.0)	1 (10.0)	1 (3.3)

^a^missing cases = 21

### Maternal mortality ratio and causes of maternal death

Amongst the 1736 women included in the study, there were a total of 1704 life births, with 5 maternal deaths, giving a maternal mortality ratio of 293 maternal deaths/100000 life births. There was one maternal death in the oxytocin only group, and this was due to myocardiac infarction. The 4 maternal deaths in the oxytocin-misoprostol group were due to HELLP syndrome (1 case), pulmonary embolism (1 case), PPH (1 case), and 1 unknown cause. Given the 51 women with PPH in the sample, the case fatality rate of PPH was 1.96%.

## Discussion

Women who received oxytocin-misoprostol were less likely to have PPH as compared to those receiving oxytocin-only (OR 0.22, 95% CI 0.08, 0.59, *p* = 0.003). There were however no significant differences in the odds of maternal death and the use of blood transfusions between the two groups: OR 3.91, 95% CI [0.44, 35.08], *p* = 0.22, and OR 0.89, 95% CI [0.14–5.63], *p* = 0.91 respectively. Sensitivity analyses showed similar results. The incidence of PPH in the Regional Hospital Bamenda was 2.9% (before adding misoprostol the incidence was 4.4% and after adding misoprostol it was 1.5%), the case fatality rate was 1.96%, and the overall maternal mortality ratio in the hospital was 293 maternal deaths/100000 life births.

Our results contrast with findings from trials conducted in Africa evaluating the use of misoprostol as an add-on to routine uterotonics in the prevention of PPH. Both Fawole et al. [22] and Hofmeyr et al. [7] did not find any significant reduction in the risk of PPH when misoprostol was used as an add-on to routine uterotonics for the prevention of PPH (relative risk [RR] 0.96, 95% CI 0.63–1.45 and RR 0.64; 95% CI, 0.38–1.07 respectively). It is however not uncommon for the results of propensity score adjusted studies to differ from that of randomised controlled trials [23, 24]. Despite the higher strength of evidence from the trials of Hofmeyr et al. [7] and Fawole et al. [22], both evaluated the use of 400 μg of misoprostol, contrary to this study which evaluated the effect of a 600 μg dose of misoprostol as an add-on to routine oxytocin. Our results therefore suggests that the dose of misoprostol used may be a determinant factor on its efficacy in reducing PPH when it is used as an add-on to oxytocin.

In Pakistan, which is a low-income country like Cameroon, Zuberi et al. [25] found 600 μg misoprostol given as an adjunct treatment for PPH to be “promising” in reducing PPH, even though they could not measure statistical significance due to a much lower than expected PPH rate. They therefore recommended its continued exploration in women with PPH [25]. Our findings support their claim, and substantiate the need for a trial evaluating the use of a 600 μg misoprostol dose for prevention of PPH.

The incidence of PPH before the intervention was 4.4%, and we believe this represents the incidence of PPH in our hospital before the start of the study. The reported overall incidence of PPH of 2.9% in this study was much lower than regional estimates of PPH of 25.7% in Africa [26]. It was also slightly lower than the 4.1% incidence of PPH found in the Yaounde University Teaching Hospital in Cameroon [27] which is a similar tertiary hospital like the Regional Hospital Bamenda. These differences may be due to differences in study settings (community vs. facility based), differences in labour care, but also the role of misoprostol in reducing PPH in our hospital. The use of an adjudication committee to make the final diagnosis of PPH from the files may equally have limited the number of cases of PPH in this study, hence the low incidence of PPH seen in this study needs to be interpreted with caution.

Given the high case fatality rate of PPH in this study (1.96%) which was higher than the < 1% case fatality rate recommended by the United Nations for women with direct obstetric complications [28], the potentially additive effect of misoprostol to curb PPH is worth considering.

The maternal mortality ratio in this study is comparable to the 287.5 per 100 000 live births reported by Tebeu et al. in a tertiary hospital in Yaounde Cameroon [29]. It is also comparable to the maternal mortality rate of 239 per 100,000 live births in low-income countries, a value which is 20 times greater than that in high-income countries [30]. Mindful of the fact that the overarching aim to curb PPH is to reduce maternal mortality, it is uncertain whether the decrease in odds of PPH with the addition of misoprostol in this study suffices to reduce the risk of maternal death. We failed to show any effect of our intervention on maternal mortality probably because due to the small numbers of maternal death, the study was underpowered to assess the effect of the intervention on this outcome. However, the observed tendency for an increased maternal mortality in the treatment group was unlikely to be due to the intervention given that the causes of these deaths were well defined, and were unlikely to be linked to the intervention.

Similarly, we failed to show any effect of our intervention on the risk of blood transfusion probably because due to the small numbers of blood transfusion especially after matching, the study was underpowered to assess the effect of the intervention on this outcome as well.

This study is limited by its design. The retrospective assessment of a natural experiment may be subject to bias, including confounding. One potential source of bias which may have played a role in the reduction of PPH in the oxytocin-misoprostol group may have been an increased awareness of the treating staff on PPH following the policy change. We had little control over this potential bias, but believe its effect would have been minimal given that the treating staffs were also not aware that a study will eventually be conducted to compare the outcome before and after the policy change.

Still in terms of bias, assessment of the outcome depended solely on hospital records which may not have been very accurate. However information from medical records for PPH and other maternal outcomes have been used as the gold standard to assess the validity of hospital discharge data [31]. We tried to minimise any potential inaccuracies in the diagnosis of PPH by the use of a blinded adjudication team of two obstetrician gynaecologists involved in the routine care of women within the said facility to assert the diagnosis based on the records.

Eight women were excluded from the study because of incomplete records. They were of similar ages as the rest of the sample, and it is unlikely that they would have differed substantially from the rest of the population in terms of other characteristics or the outcome. We therefore suspect that the effect of their exclusion would have been minimal to cause any form of selection bias.

The propensity score matching creates a new often smaller but more balanced data set in which exposed and unexposed participants have a similar distribution of covariates. It was intended as the primary analytical approach in this study. Loss of some data is a limitation of all matching techniques, but the benefits of confounding control outweigh this limitation.

Despite the propensity score matching we used to minimise any potential confounding, this method can only adjust for known confounders which have been measured [32]. However, information on other potential confounders such as a maternal history of postpartum haemorrhage, polyhydramnios, and the duration of labour were not available in the records, and were therefore not included in the propensity score model. These may constitute sources of residual confounding, and limit the strength of the causal relationship between the intervention and the outcome in the study. Another potential source of residual confounding was the birth weight of the babies for which we did not achieve balance in the propensity score model (standardised difference 31.7%). This variable was included in the multivariable regression analysis and found non-significant (result not shown).

A further limitation of this study is the fact that we were not able to assess side effects of misoprostol. Consequently, any potential benefits of misoprostol seen in the study must be taken cautiously given that side-effects such as severe hyperthermia may be life-threatening [2]. The study is equally limited by the fact that maternal deaths occurring after hospital discharge till 42 days post-delivery were unlikely to be found in the records and therefore not included in the study.

The strengths of our study include the unique ability to evaluate a natural experiment where oxytocin-misoprostol was routinely introduced, and the ability to compare within a short time frame. Other study strengths include the large sample size and the rigor in statistical analysis. Our findings were robust to extensive sensitivity analysis regarding the methods for propensity score matching analysis associated with a multivariable logistic regression analysis.

The implication of our findings is that there is a potentially beneficial effect in the use of 600 μg misoprostol as an adjunct to oxytocin to reduce PPH. While our findings may not be immediately generalizable given the potential for unknown confounding, our study provides a rationale to explore a 600 μg dose of misoprostol as an add-on to routine uterotonics for the prevention of PPH in this setting, preferably using a randomised controlled trial. If this hypothesis is backed by future research, it could lay the foundation for generalizability and advocacy for an evidenced-based policy change in the management of PPH especially in low-income settings.

## Conclusions

Our evidence suggests that using 600 μg misoprostol as an add-on to oxytocin in the prevention of post-partum haemorrhage significantly reduces the odds of PPH. This conclusion should be interpreted cautiously given the overall limitations of the study design. Further research is necessary to evaluate the net benefit of this combination in preventing PPH and consequently maternal death.

## Data Availability

Supporting data and the programming code for propensity score matching in R is available upon request from the authors.

## References

[1] Ngwenya S (2016). Postpartum hemorrhage: incidence, risk factors, and outcomes in a low-resource setting. Int J Women's Health.

[2] Chong YS, Su L-L (2006). Misoprostol for preventing PPH : some lessons learned. Lancet..

[3] Gulmezoglu AM, Souza JP, Mathai M. WHO recommendations for the prevention and treatment of postpartum Haemorrhage. Geneva: World Health Organization; 2012. p. 1–48. Available from: https://apps.who.int/iris/bitstream/handle/10665/75411/9789241548502_eng.pdf;jsessionid=5CE1B268B5103CE7C18D7EE66E8110E1?sequence=1. Accessed 10 Dec 2018.

[4] Walraven G, Dampha Y, Bittaye B, Sowe M, Hofmeyr J (2004). Misoprostol in the treatment of postpartum haemorrhage in addition to routine management: a placebo randomised controlled trial. BJOG An Int J Obstet Gynaecol..

[5] Elbohoty AEH, Mohammed WE, Sweed M, Bahaa Eldin AM, Nabhan A, Abd-El-Maeboud KHI (2016). Randomized controlled trial comparing carbetocin, misoprostol, and oxytocin for the prevention of postpartum hemorrhage following an elective cesarean delivery. Int J Gynaecol Obstet.

[6] Pakniat H, Khezri MB (2015). The effect of combined oxytocin-misoprostol versus oxytocin and misoprostol alone in reducing blood loss at cesarean delivery: a prospective randomized double-blind study. J Obstet Gynaecol India.

[7] Hofmeyr GJ, Fawole B, Mugerwa K, Godi NP, Blignaut Q, Mangesi L (2011). Administration of 400 mug of misoprostol to augment routine active management of the third stage of labor. Int J Gynaecol Obstet.

[8] World Bank. Cameroon. 2017. Available from: http://pubdocs.worldbank.org/en/819481492188154977/mpo-cmr.pdf. Accessed 10 Dec 2018.

[9] Halle-Ekane GE (2016). Use of the Partogram in the Bamenda Health District , north-west region , Cameroon : a cross-sectional study. Gynecol Obstet Res Open J.

[10] World Health Organization (WHO). International classification of diseases and related health problems. 10^th^ revision, vol. 2. Geneva: World Health Organization; 2011. Available at https://www.who.int/classifications/icd/ICD10Volume2_en_2010.pdf. Accessed 10 Dec 2018.

[11] Chandraharan E, Krishna A. Diagnosis and management of postpartum haemorrhage. Bmj. 2017;3875(September):j3875. Available from: http://www.bmj.com/lookup/doi/10.1136/bmj.j3875. Accessed 10 Dec 2018. 10.1136/bmj.j3875 28954732

[12] Briley A, Seed PT, Tydeman G, Ballard H, Waterstone M, Sandall J (2014). Reporting errors, incidence and risk factors for postpartum haemorrhage and progression to severe PPH: a prospective observational study. BJOG An Int J Obstet Gynaecol.

[13] IBM Corp (2011). IBM SPSS Statistics for Windows, Statistical Package for the Social Sciences.

[14] Team RC (2018). R: a language and environment for statistical computing.

[15] Austin PC (2011). An introduction to propensity score methods for reducing the effects of confounding in observational studies. Multivariate Behav Res.

[16] Lohr PA, Starling JE, Aiken ARA, Scott JG (2018). Simultaneous compared interval medical abortion regimens where home use is restricted. Obstet Gynecol.

[17] Randolph JJ, Falbe K, Manuel AK, Balloun JL (2014). A step-by-step guide to propensity score matching in R. Pract Assessment Res Eval.

[18] Hill J, Reiter JP (2006). Interval estimation for treatment effects using propensity score matching. Stat Med.

[19] Hosmer DW, Lemeshow S, Sturdivant R (2013). Applied logistic regression.

[20] Thoemmes F, Ong AD (2016). A primer on inverse probability of treatment weighting and marginal structural models. Emerg Adulthood.

[21] Biondi-Zoccai G, Romagnoli E, Agostoni P, Capodanno D, Castagno D, D’Ascenzo F (2011). Are propensity scores really superior to standard multivariable analysis?. Contemp Clin Trials.

[22] Fawole AO, Sotiloye OS, Hunyinbo KI, Umezulike AC, Okunlola MA, Adekanle DA (2011). A double-blind, randomized, placebo-controlled trial of misoprostol and routine uterotonics for the prevention of postpartum hemorrhage. Int J Gynaecol Obstet.

[23] Zhang Z, Ni H, Xu X (2014). Do the observational studies using propensity score analysis agree with randomized controlled trials in the area of sepsis ?. J Crit Care.

[24] Dahabreh IJ, Sheldrick RC, Paulus JK, Chung M, Varvarigou V, Jafri H (2012). Do observational studies using propensity score methods agree with randomized trials ? A systematic comparison of studies on acute coronary syndromes. Eur Heart J.

[25] Zuberi NF, Durocher J, Sikander R, Baber N, Blum J, Walraven G (2008). Misoprostol in addition to routine treatment of postpartum hemorrhage: a hospital-based randomized-controlled trial in Karachi, Pakistan. BMC Pregnancy Childbirth.

[26] Calvert Clara, Thomas Sara L., Ronsmans Carine, Wagner Karen S., Adler Alma J., Filippi Veronique (2012). Identifying Regional Variation in the Prevalence of Postpartum Haemorrhage: A Systematic Review and Meta-Analysis. PLoS ONE.

[27] Tebeu PM, Fezeu LY, Ekono MR, Kengne Fosso G, Fouelifack Ymele F, Fomulu JN (2013). Postpartum hemorrhage at Yaoundé University hospital, Cameroon. Int J Gynecol Obstet.

[28] Paxton A, Bailey P, Lobis S (2006). The United Nations process indicators for emergency obstetric care: reflections based on a decade of experience. Int J Gynecol Obstet.

[29] Pierre-Marie T, Gregory HE, Maxwell DI, Robinson EM, Yvette M, Nelson FJ (2015). Maternal mortality in Cameroon: A university teaching hospital report. Pan Afr Med J.

[30] World Health Organisation (WHO). Trends in maternal mortality: 1990–2015: estimates from WHO, UNICEF, UNFPA, World Bank Group and the UnitedNations population division: executive summary. 2015; Available at https://apps.who.int/iris/bitstream/handle/10665/194254/9789241565141_eng.pdf?sequence=1. Accessed 10 Dec 2018.

[31] Chantry AA, Deneux-Tharaux C, Cans C, Ego A, Quantin C, Bouvier-Colle MH (2011). Hospital discharge data can be used for monitoring procedures and intensive care related to severe maternal morbidity. J Clin Epidemiol.

[32] Kuss O, Blettner M, Börgermann J (2016). Propensity Score: an Alternative Method of Analyzing Treatment Effects. Dtsch Arztebl Int.

